# The Increased Risk of Road Crashes in Attention Deficit Hyperactivity Disorder (ADHD) Adult Drivers: Driven by Distraction? Results from a Responsibility Case-Control Study

**DOI:** 10.1371/journal.pone.0115002

**Published:** 2014-12-23

**Authors:** Kamal El Farouki, Emmanuel Lagarde, Ludivine Orriols, Manuel-Pierre Bouvard, Benjamin Contrand, Cédric Galéra

**Affiliations:** 1 ISPED, Centre INSERM U897-Epidemiologie-Biostatistique, University of Bordeaux, Bordeaux, France; 2 INSERM, Equipe Prévention et prise en charge des traumatismes, ISPED, Centre INSERM U897-Epidemiologie-Biostatistique, Bordeaux, France; 3 Department of Child and Adolescent Psychiatry, Charles Perrens Hospital, University of Bordeaux, Bordeaux, France; University of Leicester, United Kingdom

## Abstract

**Background and Objective:**

Both distractions (external and internal) and attention-deficit/hyperactivity disorder (ADHD) are serious risk factors for traffic crashes and injuries. However, it is still unknown if ADHD (a chronic condition) modifies the effect of distractions (irregular hazards) on traffic crashes. The objective of this study was to assess the effects of distractions and ADHD on traffic crash responsibility.

**Methods:**

A responsibility case-control study was conducted in the adult emergency department of Bordeaux University Hospital, France. Subjects were recruited among drivers injured in a motor vehicle crash between April 2010 and August 2011. Responsibility levels were estimated using a standardized method. Frequencies of exposures were compared between drivers responsible and drivers not responsible for the crash. Independent risk factors were identified using a multivariate logistic regression including test interactions between distractions and ADHD.

**Results:**

A total of 777 subjects were included in the analysis. Factors associated with responsibility were distraction induced by an external event (adjusted OR (aOR)  = 1.47; 95% confidence interval (CI) [1.06–2.05]), distraction induced by an internal thought (aOR = 2.38; CI: [1.50–3.77]) and ADHD (aOR = 2.18 CI: [1.22–3.88]). The combined effect of ADHD and external distractions was strongly associated with responsibility for the crash (aOR = 5.79 CI: [2.06–16.32]). Interaction assessment showed that the attributable proportion due to the interaction among participants with both exposures was 68%.

**Discussion:**

Adults with ADHD are a population at higher risk of being responsible for a road traffic crash when exposed to external distractions. This result reinforces the need to diagnose adult ADHD and to include road safety awareness messages delivered by the physician. Developing advanced driver assistance systems devoted to the management of attention lapses is also increasingly relevant for these drivers.

## Introduction

Injuries due to traffic accidents will become the fifth leading cause of death by 2030 if preventive interventions are not implemented quickly (World Health Organization). Whereas rising motorization in newly industrialized countries increases the worldwide population at risk for traffic crash, high income countries have faced a plateauing of the number of lives saved [1].

Among human factors related to traffic crashes, attention issues have been largely ignored by road safety research. Yet, while driving, the driver is submitted to several stimuli causing distractions to deal with. This may degrade the driver's performance in his main task and increase the risk of accidents (e.g. look diversion, slower reaction time…). Interestingly, distractions (both external and internal) and attention-deficit/hyperactivity disorder (ADHD) have been associated with traffic crashes [2]–[4]. External distractions (defined as the diversion of attention away from activities critical for safe driving, towards a competing activity) may result in insufficient attention to secure driving [5], [6]. Of note, it is suggested that external distractions could increase due to the growing use of information technologies while driving (i.e. cell phones, GPS devices). It is supported by experimental studies observing participants' behaviors when driving an instrumented vehicle with induced distracting tasks which showed poorer driving performance [7]–[9]. Recently a naturalistic study concluded that secondary-task distraction was a contributing factor in over 22% of all crashes and near crashes [10].

Internal distraction includes all signals from the body which divert the driver's attention from the driving task. It comprises mind wandering (i.e. thoughts unrelated to the task at hand) [11]–[14] which tends to occur when drivers are pre-occupied by their thoughts and can be responsible for attention lapses that might dangerously distract them from the road. In a previous analysis of the same data, we focused on mind wandering and showed that participants who declared they had experienced mind wandering with highly distracting content just before the crash had twice the risk of being responsible for their accident compared with participants who did not experience mind wandering [3].

Attention-deficit hyperactivity disorder (ADHD) is a lifelong disorder which has also been associated with traffic crashes [15]. It is a developmental disorder characterized by poor sustained attention, distractibility, impaired impulse control and hyperactivity [16]. Its prevalence in adulthood is relatively high, at around 2.5% [17]–[19]. ADHD is associated with a large range of negative outcomes comprising psychiatric disorders, socio-adaptive problems and difficulties related to daily attention failures [20]. A meta-analysis of observational studies suggests that ADHD patients receive more tickets for traffic violations, have worse driving habits and are more involved in road accidents in comparison with control groups [4]. This was described as related to the fact that they are more prone to anger on the road (road rage), aggressiveness, impulsivity, risk-taking and higher consumption of alcohol and drugs while driving. Reimer and colleagues (2010) have explored the impact of secondary tasks on ADHD drivers using a driving simulator. Their results suggested an impact on ADHD drivers ability to allocate their attention resources since they had greater difficulty performing in situations where the driving task did not demand or engender a high level of attention (such as on highway sections) [21]. Nevertheless, although impaired ability to concentrate and to sustain attention when dealing with several cognitive activities is well known, few observational studies have explored the impact of distractions on drivers with ADHD [22].

Overall, both distractions and ADHD impact driving. Whereas distractions occur irregularly and randomly, ADHD is a chronic condition characterizing the individual. We hypothesized that ADHD would modify the effect of distractions on traffic crashes. The objective of the study was to assess the effect of distractions (external and internal) and ADHD on responsibility for traffic crash and test the interactions between these two risk factors.

## Methods

### Setting

We performed a comparative study of responsibility in a population of patients involved in injurious road traffic crashes. Its principle was to compare the frequency of exposures (ADHD, external distraction and internal distraction) and confounders between drivers responsible for the crash (cases) and drivers not responsible for the crash (controls). The study was conducted in the adult emergency department of the Bordeaux University Hospital (France), which serves urban and rural populations of an area comprising more than 1.4 million people. Patients were recruited from April 2010 to August 2011. Data were collected by trained research interviewers through direct interviews using a standardized questionnaire including questions about the crash, the patient's characteristics and the potentially distracting tasks or events at the time of the crash. To preserve intimacy and improve report reliability, the part of the questionnaire with psychiatric screening tools was self-reported. All participants were informed and signed a written consent form. This study was approved by the French data protection authority (Commission Nationale Informatique et Libertés) and the regional ethics committee (Comité de Protection des Personnes Sud-Ouest et Outre Mer III).

### Participants

Patients were eligible for study inclusion if they had been admitted to the emergency department in the previous 72 hours for injury linked to a road traffic crash, were aged 18 or older, were drivers in the crash and were able to answer the interviewer (Glasgow coma score 15 at the time of interview, as determined by the attending physician). We assessed 1,436 patients for eligibility. Of these, 368 were excluded for ineligibility (93 were not the driver, 29 were admitted for more than 72 hours and 246 were unable to answer). This resulted in 1068 eligible patients. Of these, 57 refused to participate and a further 56 were excluded from the analysis because of insufficient data. Of the 955 drivers included, 178 did not answer the screening tools for psychiatric disorders. The final sample for analysis comprised 777 participants (73% of the eligible drivers). There was no significant difference between the respondent group and the non-respondent group with respect to responsibility in the crash (respectively 46.1% and 53.4% were responsible for their crash, p = 0.08).

### Measures

#### Outcome variable: responsibility for the crash

Responsibility levels in the crash were determined by a standardized method adapted from the quantitative Robertson and Drummer crash responsibility instrument [23]. Robertson and Drummer's method was validated in several studies assessing the association between responsibility and exposure to drugs. Adaptation of the method to the French context has been validated and described in previous research [24]–[27].

Notably, this method of determining the driver's crash responsibility was compared with an independent expert responsibility evaluation, achieving fair agreement with a kappa of 0.71. The method takes into consideration 6 different mitigating factors considered to reduce driver responsibility: road environment, vehicle-related factors, traffic conditions, type of accident, traffic rule obedience and difficulty of the driving task. The adapted method we used has been described elsewhere [3]. Drivers who were assigned any degree of crash responsibility were considered to be cases; drivers who were judged not responsible served as controls. The interviewers were blind to the participant responsibility status and to the psychiatric screening results when using questionnaire sections related to potential distractions, because responsibility score and psychiatric screening scores were computed during the analysis. Furthermore, compliance with traffic rules was reported after the distraction section and the psychiatric screening tools part of the questionnaire was self-administered.

#### Exposure to distractions

When interviewed, patients were asked to describe distracting events and activities that occurred just before the crash (i.e. were going on at the time of the driving mistake (inappropriate maneuver, failure to detect a threat, …) that led to the crash), from a list of potential distracting events and activities including: listening to the radio, cell phone use, navigation system use, having a conversation with passengers, eating, drinking, smoking, picking up an object, being distracted by an event outside the vehicle,…. The external distraction variable was then coded as a dichotomous variable: presence of external distraction (when there is at least one distraction reported) vs absence of external distraction.

To assess mind wandering, patients were asked to describe their thought content just before the crash. To reduce memory bias and halo effect, they were given two opportunities to report their thoughts during the interview. Two researchers (CG and EL) examined and recoded each verbal reporting of thoughts until a consensus was found. Each thought was classified in one of the following categories: thought unrelated to the driving task or to the immediate sensory input, thought related to the driving task, no thought or no memory of any thought. To capture the intensity of the thought, the participant filled in a numerical scale (0–10) answering the question: “How much did the thought disrupt/distract you?” We then dichotomized the score (slightly disrupting/distracting 0–4 vs highly disrupting/distracting 5–10). Mind wandering was then coded as a dichotomous variable: internal distraction (ID) (mind wandering with highly distracting content unrelated to the driving task or to the immediate sensory input) vs no ID (mind wandering with little disrupting/distracting content unrelated to the driving task or to the immediate sensory input or no mind wandering reported).

Potential confounders included patient's characteristics (age, sex, socioeconomic category), crash characteristics (season, time of day, location, vehicle type), and self-reported psychotropic medicine use in the preceding week Patients were also asked how many hours they had slept during the previous 24 hours. They were considered as sleep deprived if they reported sleeping less than six hours. Emotional valence was evaluated with the self-assessment manikin (SAM) tool to characterize the driver's emotional valence state just before the crash [28]. It was recoded in a dichotomous variable (negative affect v positive or neutral affect). Finally, the participants were questioned about alcohol consumption within the six preceding hours.

#### Psychiatric disorders

We investigated three psychiatric dimensions using screening tools included in a self-administered questionnaire: ADHD symptoms, depressive disorders and anxiety disorders. These two last disorders were assessed as potential cofounders since ADHD is frequently associated with anxiety disorders, mood disorders and earlier onset of major depression [20]. The Adult ADHD Self-Report Scale V1.1 (ASRS-V1.1) from the WHO Composite International Diagnosis Interview was used for ADHD screening. It examines symptoms described by the DSM-IV as being commonly seen in adult ADHD [29].

The Achenbach System of Empirically Based Assessment (ASEBA) was used to screen for depressive disorders and anxiety disorders [30]. This questionnaire is used to explore several dimensions of psychiatric disorders, referring to the classification of psychiatric disorders as defined in the DSM. We used 13 items for depressive disorders and 7 items for anxiety disorders.

Participants were asked to report any current use of psychotropic medications from a list of psychiatric disorders, impairments and symptoms (anxiety, depression, ADHD, epilepsy, insomnia, other disorder, other medication).

### Statistical analyses

Participants of the study sample were compared with the group of those excluded because they did not respond to the psychiatric disorders screening part of the questionnaire. Univariate and multivariate analyses (based on logistic regression using SAS statistical software package version 9.3–SAS Institute Inc., Cary, NC, USA) were conducted to determine associations between exposures, potential confounders and the driver's responsibility for a road traffic crash (responsible versus not responsible). Significant variables (p<0.2, univariate logistic regression) were included in a multivariate logistic regression model. We then performed a manual stepwise backward logistic regression to select the variables in the final model used below to assess interaction between distraction and ADHD. To determine the extent to which ADHD modified associations between distractions and responsibility, we evaluated interaction on an additive scale. We assessed departures from additive effects as described by Rothman and colleagues when considering interaction, particularly in the public health field [31]. It examines the extent to which the effect of both risk factors when present jointly departs from the sum of their independent effects. Interaction was assessed separately for ADHD and external distraction on the one hand and for ADHD and mind wandering on the other hand. Two separate models were fitted for each distraction with four dummy variables representing groups of exposure: no ADHD and no distraction (reference group), ADHD only, distraction only, both ADHD and distraction. For each model, joint effects of ADHD and distraction were estimated using relative excess risk due to interaction (RERI) and attributable risk proportion due to interaction (AP) and their respective 95% confidence intervals. RERI is an estimate of excess risk that is directly attributable to the interaction between two exposures. These measures of interaction were calculated using the following formulas:

RERI  =  OR_++_−OR_+ −_−OR_− +_+1 and AP =  RERI/OR_++_ (where OR designates odds ratio and the subscripts represent the presence or absence of each of the two risk factors). Bootstrapped confidence intervals were derived via 1,000 bootstrap replications of the original data. Confidence intervals were used to interpret interactions: RERI = 0 or AP = 0 means no interaction (exact additive affect); RERI>0 or AP>0 means positive interaction (beyond additive effect); RERI<0 or AP<0 means negative interaction (antagonism).

## Results

**Figure 1:**
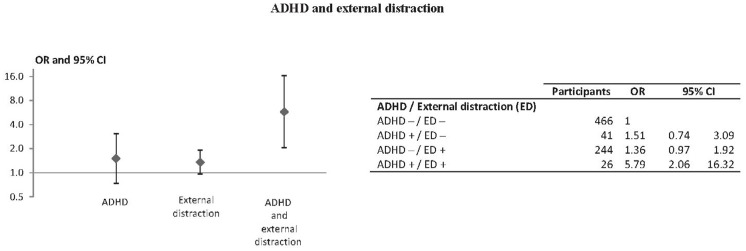
Association of ADHD and external distraction with driver responsibility, multivariate logistic regression (n = 777). Adjusted for internal distraction, alcohol use, psychotropic drug use, sleep deprivation, professional driver, gender, age, depressive disorders and anxiety disorders. (+) and (−) represent presence or absence of the risk factor.

**Figure 2:**
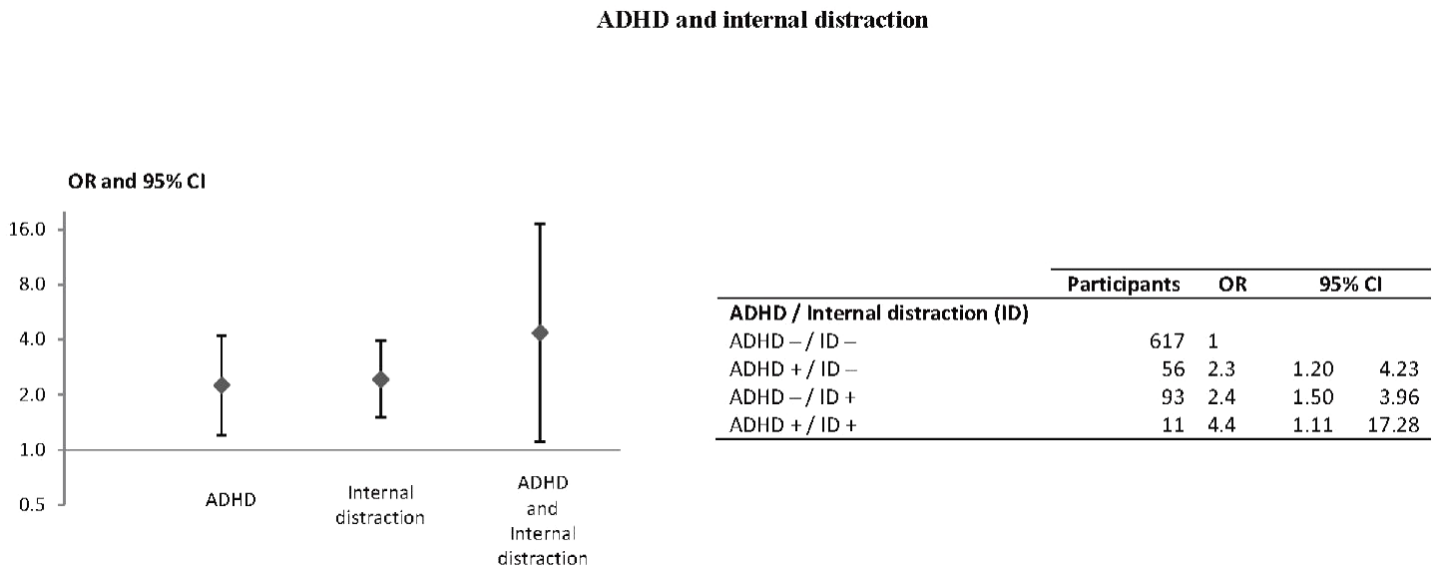
Association of ADHD and internal distraction with driver responsibility, multivariate logistic regression (n = 777). Adjusted for external distraction, alcohol use, psychotropic drug use, sleep deprivation, professional driver, gender, age, depressive disorders and anxiety disorders. (+) and (−) represent presence or absence of the risk factor.

Of the 777 subjects with complete data, 419 (53.9%) were classified as controls and 358 (46.1%) as cases. Table 1 shows the participants' characteristics and univariate associations with responsibility. The ASRS screening tool defined a group of 67 participants (8.6%) with ADHD symptoms. Depressive disorder and anxiety disorder scores were grouped into quartiles (first quartile includes the lowest scores and fourth quartile the highest). An external distraction was reported by 270 (34.8%) subjects and internal distraction by 104 (13.4%). Although depressive and anxious disorders were not significantly associated with responsibility, we forced these two variables in our final model as potential confounders. Multivariate modeling showed the following factors as significantly associated with an increased risk for being responsible for the crash (Table 2): ADHD (aOR = 2.18; 95%CI [1.22–3.88]), external distraction (aOR = 1.47; 95%CI [1.06–2.05]) and internal distraction (aOR = 2.38; 95%CI [1.50–3.77]). Alcohol consumption and psychotropic drug use were also associated with an increased risk of being responsible (respectively aOR = 3.19; 95%CI [1.70–6.01] and aOR = 2.10; 95%CI [1.23–3.60]) as well as sleep deprivation (aOR = 1.88; 95%CI [1.09–3.25]). Being a professional driver was associated with a decreased risk of being responsible (aOR = 0.58; 95%CI [0.38–0.90]). The fit of the model was good (Hosmer-Lemeshow test p = 0.46). Figs. 1 and 2 show adjusted odds ratios used to assess interactions between ADHD and external and internal distraction, respectively. There was a higher risk of being responsible for participants who had both ADHD and ED (aOR = 5.79; 95%CI [2.06–16.32]) compared with the reference group. The ADHDxED interaction was significant (RERI = 3.92; 95%CI [0.36–34.2]; AP = 0.68; 95%CI [0.13–0.95]). Regarding the ADHDxID interaction, results showed higher risks of being responsible for participants who had only ADHD (aOR = 2.26 [1.20–4.23]), for those who were exposed to ID only (aOR = 2.43 [1.50–3.96]) and also for those who were exposed to both ADHD and ID (aOR = 4.37 [1.11–17.28]). There was no significant interaction (RERI = 0.68; 95%CI [−3.68–5.4×10^6^], AP = 0.16; 95%CI [−6.88–0.99]) (Fig. 2).

**Table 1:** Sample characteristics and univariate analysis of driver responsibility for road traffic crashes, univariate logistic regression (n = 777).

	not responsible		responsible				
	(n = 419)		(n = 358)				
	n	%	n	%	OR	95% CI	p-value
**ADHD**							<10^−3^
No	396	55.8	314	44.2	1		
Yes	23	34.3	44	65.7	2.41	[1.43–4.08]	
**Depressive disorders**							<10^−2^
Quartile 1	94	63.5	54	36.5	1		
Quartile 2	84	59.2	58	40.9	1.2	[0.75–1.93]	
Quartile 3	137	51.9	127	48.1	1.61	[1.07–2.44]	
Quartile 4	104	46.6	119	53.4	1.99	[1.30–3.05]	
**Anxiety disorders**							0.66
Quartile 1	91	58.3	65	41.7	1		
Quartile 2	123	53.5	107	46.5	1.22	[0.81–1.84]	
Quartile 3	97	52.4	88	47.6	1.27	[0.83–1.95]	
Quartile 4	108	52.4	98	47.6	1.27	[0.84–1.93]	
**External distraction**							0.02
No	289	57,0	218	43,0	1		
Yes	130	48.1	140	51.9	1.43	[1.06–1.92]	
**Internal distraction**							<10^−4^
No	383	56.9	290	43.1	1		
Yes	36	34.6	68	65.4	2.5	[1.62–3.84]	
**Alcohol use**							<10^−4^
No	404	56.7	309	43.3	1		
Yes	15	23.4	49	76.6	4.27	[2.35–7.76]	
**Professional driver**							0.04
No	343	52.4	312	47.6	1		
Yes	76	62.3	46	37.7	0.67	[0.45–0.99]	
**Location**							0.06
Rural or suburban	180	50.3	178	49.7	1		
Urban	239	57,0	180	43,0	0.76	[0.57–1.01]	
**Psychotropic drug use**							<10^−2^
No	389	55.9	307	44.1	1		
Yes	30	37,0	51	63,0	2.15	[1.34–3.46]	
**Sleep deprivation**							<10^−3^
No	394	56,0	310	44,0	1		
Yes	25	34.3	48	65.8	2.44	[1.47–4.05]	
**Age (years)**							0.58
18–24	91	48.9	95	51.1	1		
25–34	116	54.2	98	45.8	0.81	[0.57–1.20]	
35–44	83	59.7	56	40.3	0.65	[0.41–1.01]	
45–54	67	54,0	57	46,0	0.82	[0.52–1.29]	
55–64	45	54.9	37	45.1	0.79	[0.47–1.33]	
≥65	17	53.1	15	46.9	0.85	[0.40–1.79]	
**Gender**							0.71
Men	238	53.4	208	46.6	1		
Women	181	54.7	150	45.3	0.95	[0.71–1.26]	
**Vehicle type**							0.20
Two-wheeler	190	51.5	179	48.5	1		
Other	229	56.1	179	43.9	0.83	[0.63–1.10]	
**Socioeconomic category**							0.79
Worker/farmer	15	46.9	17	53.1	1		
Self-employed	26	57.8	19	42.2	0.66	[0.26–1.61]	
White collar	230	55.6	184	44.4	0.71	[0.34–1.45]	
Middle management	38	53.5	33	46.5	0.77	[0.33–1.77]	
Top management/professional	18	51.4	17	48.6	0.83	[0.32–2.18]	
Unemployed	45	55.6	36	44.4	0.71	[0.31–1.60]	
Student	47	47.5	53	52.5	0.98	[0.44–2.17]	
**Usual route**							0.10
No	76	48.1	82	51.2	1		
Yes	343	55.4	276	44.6	0.75	[0.53–1.06]	
**Season**							0.04
Summer	94	48.2	101	51.8	1		
Autumn	112	55.2	91	44.8	1.32	[0.89–1.96]	
Winter	71	65.1	38	34.9	0.66	[0.41–1.07]	
Spring	142	52.6	128	47.4	1.11	[0.77–1.60]	
**Time of the day**							0.48
2000-0459	127	52.1	117	48,0	1		
0500-1959	292	54.8	241	45.2	0.90	[0.66–1.21]	
**Negative affect**							0.03
No	349	55.8	276	44.2	1		
Yes	70	46.1	82	54	1.48	[1.04–2.11]	

**Table 2:** Factors associated with driver responsibility for road traffic crashes, multivariate logistic regression (n = 777).

	Participants	OR	95% CI	p value
**ADHD**				<10^−2^
No	710	1		
Yes	67	2.18	[1.22–3.88]	
**External distraction**				0.02
No	507	1		
Yes	270	1.47	[1.06–2.05]	
**Internal distraction**				<10^−3^
No	673	1		
Yes	104	2.38	[1.50–3.77]	
**Alcohol use**				<10^−3^
No	713	1		
Yes	64	3.19	[1.70–6.01]	
**Psychotropic drug use**				<10^−2^
No	696	1		
Yes	81	2.10	[1.23–3.60]	
**Sleep deprivation**				0.02
No	704	1		
Yes	73	1.88	[1.09–3.25]	
**Professional driver**				0.02
No	655	1		
Yes	122	0.58	[0.38–0.90]	
Gender				0.28
Men	446	1		
Women	331	0.84	[0.60–1.16]	
**Age (years)**				0.56
18–24	186	1		
25–34	214	0.83	[0.55–1.26]	
35–44	139	0.64	[0.40–1.04]	
45–54	124	0.78	[0.48–1.29]	
55–64	82	0.73	[0.41–1.28]	
≥65	32	0.62	[0.27–1.44]	
**Depressive disorders**				0.16
Quartile 1	148	1		
Quartile 2	142	1.05	[0.63–1.75]	
Quartile 3	264	1.47	[0.91–2.39]	
Quartile 4	223	1.74	[0.99–3.08]	
**Anxiety disorder**				0.07
Quartile 1	156	1		
Quartile 2	230	0.97	[0.61–1.53]	
Quartile 3	185	0.88	[0.52–1.47]	
Quartile 4	206	0.53	[0.29–0.95]	

Among the 777 subjects included in the analysis, only 1 participant declared to use ADHD medication. Additional sensitivity analysis was performed and the results remained consistent when this participant was not included.

## Discussion

### Principal findings and interpretation

Participants who had symptoms of ADHD, and those exposed to external/internal distractions were significantly more likely to be responsible for a road crash after adjustment for a range of potential confounders. Assessment of interaction showed a higher risk of being responsible when exposed to both external distraction and ADHD. The proportion of risk that is attributable to the interaction for those who were exposed to both ADHD and external distraction was 68%. These results are consistent with the increased risk of road crashes described in patients with ADHD. We can hypothesize that the effect of external distraction on the risk of being responsible for a road accident was higher in participants with ADHD compared with controls because of greater difficulty in managing the dual task situation. The process of managing the dual task involves the subject's ability to allocate attention flexibly between the tasks to be performed, an ability which can be affected by working memory impairment. Hence, the interaction between ADHD and external distraction could be related to the frequent working memory impairments in ADHD [32], [33]. Working memory is defined as a cognitive system that provides temporary storage and manipulation of the information necessary for complex cognitive tasks [34]. Working memory impairments may be responsible for difficulties completing two tasks simultaneously (dual task) or resisting an external distraction. External distractions may compete with the main task of driving and involve the driver in a dual task situation. This multitasking overload can affect the driver's performance through a large range of inattention-related impairments, such as failing to detect stimuli in the periphery of the visual field, fewer rear-view mirror and speedometer checks, increased time reaction [35]. The more the secondary task calls upon cognitive processing resources, the more it alters the driver's behavior. Moreover, attention ensures consistency of behavior towards a purpose (top-down) and allows a quick response to changes in the environment (bottom-up). It is therefore important for a driver distracted by secondary information (indicating a situation of danger) to process it quickly and to return to the main task. The interaction between cognitive flexibility and resistance to distraction allows this process to move back and forth between main and secondary task stimuli. These cognitive abilities involve the working memory, which has to stick to the purpose of the main task. Thus, because of a higher susceptibility to being distracted by surrounding stimuli, the same external distraction can capture the attention of a driver with ADHD and cause greater attention failures than in a control driver, resulting in a greater increase in the risk of being responsible for an accident.

In our sample, only 1 participant declared to use ADHD medication. This is consistent with the fact that ADHD is currently underdiagnosed and treated in France and many European countries [36]. Given that the use of psychotropic stimulants improves ADHD impairments, additional sensitivity analysis was performed. The results remained consistent when this participant was not included.

### Strengths and weaknesses

To our knowledge, there is no study of the interactions between distractions and ADHD, except for driving simulator studies [22]. The large sample size of the present observational study provided the first approach to real driving conditions. The standardized method using the occurrence conditions, the environment and vehicle-related factors to define responsibility for each driver is particularly useful when studying the impact of human factors by looking for an over-representation of these factors in the responsible group. Assessment of interaction using an additive scale allows the proposal of recommendations whose impact is measurable.

This study has limitations which should be taken into account when interpreting its results. It includes subjective evaluation of both impairments and distraction exposures. The assessment of psychiatric disorders was not based on clinical diagnosis. Reassuringly, the ASRS-V1.1 has performed well in studies, shows good internal consistency, and good concordance with clinical diagnosis (area under the receiver operator curve 0.90) [29], [37]. Distractions were assessed retrospectively and can be affected by a possible memory bias. We can, however, assume this potential bias to be non-differential among cases and controls. For external distractions, it is possible that responsible drivers tended to under-report their distractive activities while driving since some of them are prohibited (namely cell phone use). This possible differential misclassification bias would then have resulted in an underestimation of the association measure. Another limitation with respect to external validity is that the participants included were not representative of all traffic crash victims. The drivers whose condition did not require emergency care, because of low crash severity, could not be recruited. Similarly, patients who were unable to answer the questions because of the severity of their condition were not included (17% of eligible patients). The non-inclusion of these two groups, corresponding to the extremes in crash severity, made it difficult to interpret the selection bias effect potentially induced by an association between responsibility and severity. It is noteworthy that no objective measures of cognitive impairments were performed in this observational study. Hence, it would be of great interest to explore our findings using computerized neuropsychological testing and driving simulators

### Implications and future research

ADHD and external distraction are known risk factors for traffic crashes. The interaction assessed on an additive scale highlighted the impact of the combination of these two factors, a result with potential consequences as regard to prevention. This suggests potential public health benefits of ADHD screening. It is estimated that 2.5% of adults have ADHD, though many remain undiagnosed [19]. Improved screening and diagnosis of ADHD in adults would enable adoption of care strategies that focus on reducing attention impairment and improving cognitive performance. These strategies, which may also include road safety awareness messages, are likely to reduce the risk of road crashes in patients with ADHD. Some authors have reported improved driving performance in ADHD patients treated pharmacologically. However, these studies were all performed on driving simulators in small numbers of participant, which limits their generalizability to real driving conditions [4], [15], [22], [38]. A recent epidemiological Swedish study has shown that the risk of serious transport crashes was significantly reduced in ADHD patients while they were treated [39]. Other approaches are also being developed based on cognitive or emotional therapy. Cognitive remediation strategies to improve executive functions seem to decrease attention impairments and to improve attention flexibility in adult patients with ADHD [40]. Among emotional therapies, meditation techniques such as "mindfulness" may also improve sustained attention [41]. These strategies were also applied to small samples and further research seems necessary. Interestingly, there is a growing literature on advanced driver assistance systems, which have become an active area of traffic safety research [42]. Such systems support information assimilation and help to avoid distraction (e.g. if the driver is visually distracted, a warning halo refocuses his or her attention). It is now considered that there is a safety potential for advanced driver assistance systems, which may also be of great value in ADHD patients.
